# Retrospective analysis of pulmonary cryptococcosis and extrapulmonary cryptococcosis in a chinese tertiary hospital

**DOI:** 10.1186/s12890-023-02578-2

**Published:** 2023-07-27

**Authors:** Jiejun Shi, Jianhua Chen, Liqing Hu, Ada Hoi Yan Ma, Haoxuan Hu, Chuwen Wang, Jiajia Huang, Qifa Song, Guoqing Qian

**Affiliations:** 1 Department of Infectious Diseases, Ningbo First Hospital, Ningbo University, Ningbo, Zhejiang Province China; 2 Department of Radiology, Ningbo First Hospital, Ningbo University, Ningbo, Zhejiang Province China; 3 Department of Clinical Laboratory, Ningbo First Hospital, Ningbo University, Ningbo, Zhejiang China; 4 Nottingham University Business School, University of Nottingham Ningbo China, Ningbo, Zhejiang Province China; 5 Department of Internal Medicine, Fenghua District Hospital, Ningbo, Zhejiang Province China; 6 School of Medicine, Ningbo University, Ningbo, Zhejiang China; 7 Medical Data Research Center, Ningbo First Hospital, Ningbo University, Ningbo, Zhejiang Province China

**Keywords:** Pulmonary cryptococcosis, Extrapulmonary cryptococcosis, Immune state, Cryptococcal antigen test

## Abstract

Cryptococcosis is an invasive fungal disease with increased morbidity in China over the past two decades. *Cryptococci* can infect immunocompromised hosts as well as immunocompetent ones. In this study, we reviewed data of 71 inpatients with cryptococcosis at Ningbo First Hospital from May 2010 to May 2020 and compared the clinical profiles of pulmonary cryptococcosis (PC) and extrapulmonary cryptococcosis (EPC). Of 71 patients (38 males, 33 females), 70 were non-HIV. The annual inpatient population increased dramatically, especially in the PC group. PC was confirmed in 77.46% (55/71) of cases by pathology. The rest were EPC including intracranial infection (15.49%, 11/71) and cryptococcemia (7.04%, 5/71). Compared with PC, a larger proportion of EPC patients were found to have immunocompromised conditions judged by predisposing factors (*p* < 0.01), or detectable humoral or cellular immunodeficiency. Fever and headache were more common in EPC patients (*p* < 0.001). Patients with EPC had lower serum sodium level (*p* = 0.041), lower monocyte counts (*p* = 0.025) and higher C-reactive protein (*p* = 0.012). In our study, the sensitivity of cryptococcus antigen detection for EPC was 100% regardless of sample type, while serum lateral flow assay (LFA) tested negative in 25% (5/20) of PC. Immunocompromised hosts are more likely to suffer from EPC than PC.

**Supplementary Information:**

The online version contains supplementary material available at 10.1186/s12890-023-02578-2.

## Introduction

Cryptococcosis is a worldwide invasive fungal disease mainly caused by seven species derived from two varieties within *Cryptococcus neoformans* (*C.neoformans*) and five genotypes within *C. gattii* [1, 2]. Decaying hollows of trees, feces of pigeons and some insects are the major ecological niches of *Cryptococci* [1, 3]. Hosts exposed to pigeon droppings can develop pulmonary cryptococcosis (PC) by inhaling aerosols containing *Cryptococci*. Cryptococcosis encompasses a wide spectrum of infections that range from localized pulmonary infection, which may resemble tuberculosis or tumor, to severe disseminated infections, including intracranial infection and cryptococcemia, which are generally referred to as extrapulmonary cryptococcosis (EPC). The most common extrapulmonary focus of infection was the central nervous system (CNS), which accounts for 83.4% of total cryptococcal infections in China [4] and global 19% of AIDS-related deaths annually [5]. Global data revealed, in patients with AIDS, cryptococcal meningitis (CM) causes approximately 625,000 deaths annually [6]. Without timely identification and therapy, the risk of long-term neurological sequelae will increase to 45% [7]. Therefore, distinguishing cryptococcal disseminated infection from localized PC is vital to improve prognosis.

To the best of our knowledge, few studies were conducted to compare the clinical profiles of PC and EPC. Hence, we conducted this study to explore underlying risk factors for cryptococcal dissemination via comparing different cryptococcal infection patterns (PC vs. EPC).

## Methods

A retrospective analysis was performed based on discharge summaries from May 2010 to May 2020 at Ningbo First Hospital which is a 1631-bed tertiary hospital in South China. The data of inpatients diagnosed with cryptococcosis were extracted from the electronic medical record system. The following information was collected: demographic data, clinical presentations, laboratory and radiological examinations, treatments and outcomes. Patients younger than 18 years old or diagnosed with cryptococcosis but lacking sufficient diagnostic evidence were excluded. All the selected patients met at least one of the following diagnostic criteria: (1). *Cryptococcus* was isolated from blood or sterile body fluid samples (such as CSF, bone marrow, pleural effusion) by culture; (2). Histopathological examinations indicated *Cryptococcus* infections; (3). CSF tested positive for Cryptococcal antigen (CrAg). (4). Those with blood culture positive for *Cryptococcus* were diagnosed with cryptococcemia [8].

### Immune state assessment

People with at least one of the following comorbidities were presumed to have immunocompromised conditions: uncontrolled diabetes mellitus, autoimmune diseases, chronic renal or hepatic diseases, malignancies, long-term usage of immunosuppressants or glucocorticoids, human immunodeficiency virus (HIV) infection, tuberculosis, and history of organ transplantation.

Some patients were tested for their immune function. The criteria for being categorized as immunocompromised state are as follows:

1. At least two of the serum immunoglobulin levels (IgG, IgA and IgM) were below the lower limit of normal reference
2. The ratio of CD4/CD8 T cells < 1 or CD4 + T lymphocyte count was < 350/µL
3. Neutrophil count < 2.0 × 10^3^/ml or lymphocyte count < 10^3^ /ml

### Histopathological examinations

Of the 55 tissue-proven pulmonary cryptococcosis patients, 32 underwent thoracoscopic surgery, 1 was conducted transbronchial lung biopsy (TBLB) and the remaining were performed on percutaneous lung puncture. One case with cryptococcal a brain abscess was confirmed by craniotomy. All the tissue blocks were fixed by paraffin, stained by hematoxylin–eosin, periodic acid–Schiff, Grocott’s methenamine silver and mucus card Red, and finally observed under microscopy. India ink test was direct microscopy of CSF in India Ink.

### Culture

The specimens (CSF, blood, bone marrow) were cultured on Sabouraud dextrose agar at 35℃ or 25℃ for 3–5 days to observe the growth of fungal colonies. The species were identified by VITEK 2 COMPACT (BIOMERIEUX, France) and matched YST identification cards.

### Cryptococcal antigen tests

CrAg tests were performed on serum or CSF using the CrAg Lateral Flow Assay (LFA) (Immuno-Mycologics, Inc. Norman, OK USA), CrAg Latex Agglutination (LA) System (CALAS; Meridian Biosciences Inc., Cincinnati, OH), and capsular polysaccharide glucuronoxylomannan (GXM) antigen test (Dynamiker Biotechnology Co., Ltd., China). Protocol can be referred to for detailed operation and result illustration.

### Radiological examinations

All the PC patients during hospitalization had at least one chest CT scan. Brain CT scan or magnetic resonance imaging (MRI) was performed on those patients with CNS symptoms. Routine CT scans were performed on a series of CT systems (Somatom Sensation 16, Siemens Medical Systems, Forchheim, Germany; Aquilion 64, Toshiba Medical Systems, Otawara, Japan; Brilliance 16, Philips Medical Systems, Amsterdam, Netherlands). Consecutive 2 to 5 mm thick sections were fetched from the lung apex throughout the base for chest CT scan. Thick section for brain CT scan is 5 mm. Window settings used for browsing lung parenchyma were at width 1400–1600 Hu, level − 550 to − 600 Hu; corresponding values for soft tissues were at width 400 Hu, level 40 Hu. Window settings used for browsing brain were at width 80 Hu, level 40 Hu. MRI was performed on Sonata 1.5T, Siemens Medical Systems, Forchheim, Germany. Brain MRI has a series of scanning sequences including T2WI, T1WI, DWI and T2WI FLAIR.

### Statistical analysis

SPSS Statistics 25 software (IBM, Armonck, NY, USA) was used to analyze the data. Numerical data were expressed as mean ± SD. Continuous variables with normal distribution were compared using Student’s t-test, and those of skewed distribution or with uncertain value at one end were analyzed using the Mann-Whitney U test. Categorical data were analyzed via the Chi-squared test or Fisher’s exact test.

## Results

Of 197 patients clinically diagnosed with cryptococcal infection, after applying the inclusion and exclusion criteria, 71 were identified and included in our study among whom only one had HIV infection. They were generally divided into PC group (55 patients confirmed by pathology) and EPC group (16 patients). Demographic information was summarized as Table 1. Among all the patients, 11 were farmers with a history of exposure to plants, but only one had a direct contact with pigeon droppings.

**Table 1:** Baseline characteristics and partial laboratory data of participants in our study

Variables	Pulmonary cryptococcosis	Extrapulmonary cryptococcosis	*P*-value
**Patients**	55	16	N/A
**Gender**			
Male	27 (49.09%)	11 (68.75%)	0.165
Female	28 (50.91%)	5 (31.25%)	
**Age** (mean ± SD)	53.45 ± 12.47	54.75 ± 18.16	
**Smoking**^e^	7	4	0.42
**Exposure history**^a^	8	4	0.55
**Preexisting immunocompromised conditions**^**b**^	13 (23.64%)	12 (75%)	0.000153
Diabetes mellitus	4	4	0.127
Autoimmune disease	5	6	0.018
Chronic renal disease	2	3	0.127
Chronic hepatic diseases	1	1	
Malignancies	4	2	0.88
Long-term usage of immunosuppressants or glucocorticoids	6	8	0.002
Immunosuppressants	2	0	
Glucocorticoids	3	1	
Immunosuppressants and glucocorticoids	1	7	
HIV infection	0	1	
Previous tuberculosis	2	0	
History of organ transplantation	0	0	
Hypertension	17	4	0.89
**Symptoms**	22 (40%)	14 (87.5%)	0.001
Fever	5	11	< 0.001
Headache	0	8	< 0.001
Respiratory symptoms^c^	22	3	0.12
**Laboratory tests**			
Na+ (mmol/L)	140.49 ± 2.64	137.77 ± 4.72	0.041^ξ^
CRP > 5 mg/L	29.4% (10/34)	64.3% (9/14)	0.025
CRP value (mg/L)	--	--	0.012^Ψ^
Monocyte counts (/uL)	459.82 ± 167.28	363.12 ± 234.61	0.025^Ψ^

^a^Exposure to avian excreta or plants or pets or working as a farmer

^b^Patients with at least one of the following comorbidities: uncontrolled diabetes mellitus, autoimmune disease, chronic renal or hepatic diseases, malignancy, long-term usage of immunosuppressants or glucocorticoids, human immunodeficiency virus (HIV) infection, tuberculosis, history of organ transplantation. Malignancy includes current malignant solid tumor or hematologic neoplasms

^c^At least one of the following presentations: cough with or without sputum, chest pain, dyspnea or hemoptysis

^e^Smoking may be a risk factor for cryptococcosis as reported by previous studies

ξ Student’s t-test

Ψ Mann-Whitney U test

**Fig. 1:**
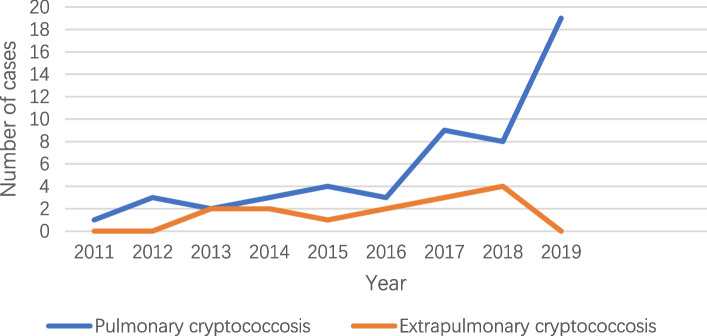
The annual cryptococcosis inpatient population from Jan 2011 to Dec 2019

**Fig. 2:**
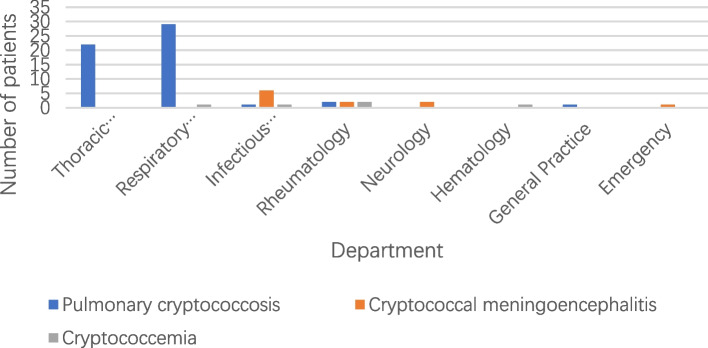
Distribution of initial admission departments of patients with cryptococcosis

Overall, an obvious rise was observed in cryptococcosis population during the period especially for PC (Fig. 1). As depicted in Fig. 2, most PC patients were initially admitted to the Department of Thoracic Surgery (22 patients, 40%) and Department of Respiratory Medicine (29 patients, 52.73%). However, the majority of EPC cases were initially brought to the Department of Infectious Diseases (7 patients, 43.75%). Among the PC cases, 32 patients were misdiagnosed as lung tumors and 15 as bacterial pneumonia at initial presentation. The time-to-correct-diagnosis for these subjects varies from 5 to 18 days. Nine cases of PC underwent lumbar puncture to exclude CM. Of the 16 EPC cases, 11 were intracranial cryptococcal infections including one cryptococcal brain abscess who underwent surgical resection, the rest 5 cases were cryptococcemia.

### Symptomatology

As summarized in Table 1, compared to EPC, more PC patients (60%, 33/55) (χ2 = 11.188, *p* = 0.001) were admitted to our hospital due to pulmonary space-occupying lesions found in physical examination which mimic malignancy. Respiratory symptoms were the most common presentations in PC, so as fever in EPC. Headache is the exclusive clinical symptom in EPC cases. We did not observe abnormal muscle strength and tone, altered mental status, impaired sensory function in our CM patients.

### Laboratory examination

Among those with intracranial cryptococcal infection, all 11 patients underwent CSF routine examinations including India ink test and culture while only 3 tested positive in both tests. The positivity of India ink test (54.5%, 6/11) was slightly higher than that of culture (45.5%, 5/11) (Table 2). In 11 patients with CM, CSF (5 patients) and serum (6 patients) lateral flow assay were all positive (Table 2), among whom four patients took both serum LFA and CSF LFA showing 100% agreement of the two tests. *Cryptococci* were isolated by blood culture in all the 5 patients with cryptococcemia and CSF culture in 45.45% (5/11) of patients with intracranial cryptococcosis. Of the 11 *Cryptococcus* strains isolated by culture (5 from blood, 1 from bone marrow, 5 from CSF), 10 were *C.neoformans* species complexes, and the remaining one was *C.laurentii*.

**Table 2:** Etiological detection tests of patients with cryptococcosis

Test items	Pulmonary cryptococcosis		Extrapulmonary cryptococcosis	
	**Surgical**	**Nonsurgical**^b^	**Intracranial cryptococcosis**	**Cryptococcemia**
**Serum LFA**	40% (2/5)	86.7% (13/15)	100% (6/6)	100% (1/1)
**CSF LFA**	--	0% (0/5)	100% (5/5)	--
**Serum LA**	100% (1/1)	100% (6/6)	--	100% (1/1)
**CSF LA**	--	--	100% (2/2)	--
**Serum GXM**	100% (1/1)	100% (7/7)	100% (2/2)	--
**CSF culture**	0% (0/1)	0% (0/2)	45.5% (5/11)	--
**India ink test**	0% (0/2)	0% (0/5)	54.5% (6/11)	--
**Pathology**	100% (32/32)	100% (23/23)	100% (1/1)	--
**Blood culture**	--	--	0 (0/10)	100% (5/5)^a^
**Bone marrow culture**	--	--	0 (0/1)	100% (1/1)^a^

All the tests were conducted before antifungal therapy or surgical resection. All the data were presented as percentage (positive population/ total detected population). Among 11 patients with intracranial cryptococcosis, 5 didn’t have chest CT scan. None of EPC patients underwent pulmonary pathological and microbiological examinations to diagnose PC. Some subjects in PC/EPC group were positive for more than one tests. Detailed data can be obtained from the first author

--: Nobody in the cohort had specified test results

GXM: capsular polysaccharide glucuronoxylomannan

^a^One patient has both blood culture and bone marrow culture positive for *Cryptococcus*

^b^Nonsurgical PC patients were generally treated in respiratory ward and diagnosed via percutaneous lung biopsy

In the PC cohort, serum LA exhibited higher positivity (100%, 7/7) than serum LFA (75%, 15/20). Two patients tested negative for serum LFA who underwent percutaneous lung biopsy prior to the LFA test.

The EPC group had significantly higher C-reactive protein (CRP) level (Mann-Whitney U test, Z=-2.503, *p* = 0.012), lower serum sodium levels (Student’s t-test, *p* = 0.041) and monocyte counts (Mann-Whitney U test, Z=-2.243, *p* = 0.025) (Table 1) than the PC group.

### Radiological findings

Of the 55 patients with PC, 45.45% (25/55) of cryptococcal lesions in were located in the right lung, followed by 32.73% (18/55) in left lung and 21.82% (12/55) bilaterally. Single nodule was found in 29 patients, multiple nodules in 9 patients, pneumonic infiltrates in 8 patients and a mixture of these morphologies in 9 patients. Notably, 86.21% (25/29) of single nodule lesions mimicked lung tumors in shape which were parenchymal or subsolid nodules with lobulations or spiculated margins, thick-walled cavities or cavities with nodular margins, pleural indentation or mediastinal invasion. Single nodule larger than 1 cm accounted for 65.52% (19/29) of the cases. Cavities (8/55), Pleural effusion (2/55) and calcification (1/55) were uncommon in PC.

Among 16 cases with EPC, 11 have chest CT scan, 7 have brain MRI, and 6 have brain CT scan. Pneumonic infiltrates were the most common chest CT findings (10/11) in EPC. Pulmonary single nodule was observed in only one case with EPC. Abnormal signals were discovered in brain MRI of 2 EPC patients and in brain CT of 1 patient.

### Immune state assessment

In 25 patients with comorbidities, EPC cases were more likely to have immunocompromised factors (Pearson χ2 = 14.334, *p* < 0.001) (Table 1). More EPC patients (4 out of 8) were detected to have impaired humoral immune than PC (0 out of 20) (Fisher’s exact test, *p* = 0.003) as shown in Table 3. Cellular immunity in terms of the ratio of lymphocyte counts less than 10^3^/ml was at a lower level in the PC cohort (7.3%) compared with EPC (81.3%) (correction for continuity χ2 = 33.296, *p* < 0.001) as shown in Table 3.

**Table 3:** Immune function evaluation between pulmonary cryptococcosis and extrapulmonary cryptococcosis

Test items	Pulmonary cryptococcosis	Extrapulmonary cryptococcosis	*P*-value
**Immunoglobulins**^**#**^	0% (0/20)	50% (4/8)	0.003
**Neutrophil count < 2.0 × 10**^**3**^**/ml**	1.8% (1/55)	12.5% (2/16)	0.125
**Lymphocyte count < 10**^**3**^**/ml**	7.3% (4/55)	81.3% (13/16)	< 0.001
**CD4/CD8 < 1**	33.3% (4/12)	60% (3/5)	0.593
**CD4 + T lymphocyte count < 350/µL**	0 (0/12)	0 (0/5)	

^#^The percentage of patients who had at least two of the serum immunoglobulin levels below the lower limit of normal reference

### Treatment and outcome evaluation

Among 32 PC patients who underwent thoracoscopic surgery due to lesions mimicking malignancy. Postoperative antifungal agents of fluconazole (21 patients) or itraconazole (1 patient) were prescribed in 22 (68.75%) patients. The remaining 23 PC patients were treated in internal medical ward among whom 19 were given standard antifungal therapies with fluconazole [9]. During hospitalization, antifungal regimen was adjusted in 3 PC patients due to the unsatisfactory therapeutic effect. During follow-up, all the PC patients achieved favorable outcomes.

Treatments of EPC patients were not standardized since 68.75% (11/16) of patients were not given amphotericin B due to its severe nephrotoxicity. 3 patients died from cryptococcemia. 6 patients with EPC were transferred to superior hospitals or gave up treatment due to the aggravated condition. The remaining 7 patients were discharged when their condition improved.

## Discussions

According to a recent national survey, cryptococcosis (7.7%) has become the second most common invasive yeast infection in China [10]. Similarly, in Europe and North America and the Latin America region, the incidences of CM have increased by approximately two-fold since 2009 [5]. Although HIV is the most common underlying factor for cryptococcosis, one retrospective analysis of data from Chinese Database indicated only 16% of patients with cryptococcosis were co-infected with HIV [4]. One single-center research revealed that HIV-positive cryptococcosis cases had been falling while the number of HIV-negative/non-transplant cryptococcosis was increasing [11]. Non-HIV CM patients have higher mortality than HIV-infected ones, which is related to T cell–mediated inflammatory injury [12]. Therefore, non-HIV cryptococcosis may be a focus of the fungal infection which motivated us to conduct this research.

Previous studies revealed *Cryptococci* mainly infected HIV-negative Chinese [4, 13] which concurred with our findings. This may be related to multiple polymorphisms in the genes encoding mannose-binding lectin (MBL) and Fc-gamma receptor 2B (FCGR2B) found in the Han population [13]. MBL is an important member of pathogen recognition receptors which is essential for activating host innate immunity. Deficient MBL production, especially in immunocompetent patients, may lead to higher risk for CM [13]. The frequencies of FCGR2A 131R/R, FCGR3B NA2/NA2, and FCGR2B 232T/T were similar among Asian populations but different from Caucasian populations. FCGR2B 232I/T genotype was revealed to be associated with CM in non-HIV individuals, though no significant association was found between other genotypes, including FCGR3B, FCGR2A and FCGR3A, and CM [14]. FcγRIIB has an immunoreceptor tyrosine-based inhibitory motif (ITIM) in its cytoplasmic domain while FCGR2B 232I/T is located in the transmembrane domain. FcγRIIB plays a role in regulating the immune system and can also recognize glucuronoxylomannan which is the major component of the capsule of *C. neoformans*. FCGR2B 232I/T transformed to FCGR2B 232T making FcγRIIB unable to interact with activating receptors and exert inhibitory effect [14] which accounts for the role of FCGR2B 232I/T and FcγRIIB in development of CM.

Annual population with PC was much greater than that of EPC in our hospital. This percentage is slightly higher than a previous report [15]. A large-scale investigation [4] based on Chinese data reported that the most common symptoms of CM were headache (87%) and fever (74%) which agrees with our findings. Unlike previous findings [16, 17], based on our study, smoking seems to pose little effect on cryptococcosis severity. Far less than that reported by Zhang et al [18], only 16.9% of our cases were recorded to have exposure history of pigeon droppings which may be partially attributed to the ignorance of doctor in charge of medical history collection.

In PC cohort, cryptococcal lesions showed priority of right lung distribution which is consistent with previous research [18]. The lesions are morphologically indistinctive and easily misdiagnosed as other pulmonary diseases.

Although the sample size was small, we find immune status is correlated with cryptococcosis severity. Lymphopenia and monocytopenia may be risk factors for cryptococcal dissemination which agrees with previous studies [19–21]. Panackal AA et al. discovered susceptibility to CM was associated with idiopathic chronic lymphopenia [19], reduced monocyte in the CSF and poor phagocytosis of fungal cells by M2 macrophage [20] which indicated dysfunction of monocyte-macrophage system.

CrAg detection methods, such as LFA, LA and enzyme immunoassay, are widely used in diagnosing cryptococcosis. Serum LFA was reported to have higher sensitivity and wider serotype coverage than LA in clinical practice [22, 23]. However, our study indicated serum LA test seems to be more sensitive to identify PC than LFA, although they share the same sensitivity in EPC. Large-scale studies are required to verify the above findings. Recently Temfack et al. conducted a meta-analysis of diagnostic test accuracy studies on CrAg in serum and CSF for detecting CM which revealed high sensitivity and specificity of CrAg detection in both serum and CSF samples of adults living with HIV [24]. Hence, the author concluded negative serum CrAg may rule out CM in those HIV patients. This is also true for non-HIV patients in our cohort. Since all the serum CrAg detections in our study, regardless of LFA test or LA or glycuronoxylomannan, had 100% positivity in EPC cases. Although our data are insufficient to establish sensitivity differences of the three CrAg detection methods between two cohort, we found about 25% of the PC cases tested negative for serum LFA test, which concurs with aforementioned studies [25]. The positive rate of serum LFA (75%) in our PC patients was higher than that previously reported (25–56%) [25].

Hamadani [21] discovered hyponatremia may be a risk factor for cryptococcal infection which was reinforced by our research. Higher serum sodium levels appear to work against cryptococcal dissemination though further studies are required to explore the underlying causation.

Some limitations exist in our study. First, number of subjects, especially for those with EPC, is not large enough to get convincing conclusions. Second, not all the participants underwent lumbar puncture to confirm or expel intracranial *Cryptococcus* infection.

## Conclusions

To sum up, we found that 98.6% of participants diagnosed with cryptococcosis in our study were HIV-negative which indicates non-HIV hosts are susceptible to *Cryptococci* as well. There were notable differences in the clinical profile between the PC and EPC groups. EPC patients were more likely to experience symptoms such as fever and headache. PC is easily misdiagnosed due to nonspecific clinical and imaging features. In this study, serum CrAg tests were found to be more reliable in diagnosing EPC than PC.

## Supplementary Information

**Additional file 1.**

## Data Availability

The original data supported this study can be looked up in our hospital’s electronic medical record system, further inquiries can be directed to the first author. Datasets are not suitable to be deposited to publicly available repositories due to patient privacy.

## Abbreviations

PC: Pulmonary cryptococcosis
EPC: Extrapulmonary cryptococcosis
LFA: Lateral flow assay
C.neoformans: Cryptococcus neoformans
CNS: Central nervous system
CM: Cryptococcal meningitis
CrAg: Cryptococcal antigen
HIV: Human immunodeficiency virus
TBLB: Transbronchial lung biopsy
LA: Latex Agglutination
GXM: Capsular polysaccharide glucuronoxylomannan
MRI: Magnetic resonance imaging
CRP: C-reactive protein
MBL: Mannose-binding lectin
FCGR2B: Fc-gamma receptor 2B
ITIM: Immunoreceptor tyrosine-based inhibitory motif

## Declarations

All data was reviewed retrospectively according to the ethical standards of the observational research. Participant names were hidden in this study.
