# Supplementary material for: Retrospective analysis of pulmonary cryptococcosis and extrapulmonary cryptococcosis in a chinese tertiary hospital
Source: BMC Pulm Med. 2023 Jul 27;23:277. doi: 10.1186/s12890-023-02578-2 (PMC10375642; doi:10.1186/s12890-023-02578-2)
Supplement: Supplementary file 1 — Additional file 1. [file 12890_2023_2578_MOESM1_ESM.docx]

Normal reference ranges of quantitative items

IgG: (751-1560) mg/dl,

IgA: (82-453) mg/dl,

IgM: (46-304) mg/dl,

neutrophil count：(1.8-6.3) * 10^9/L,

lymphocyte count: (1.1-3.2) * 10^9/L,

ratio of CD4/CD8 T cells: 0.89-2.01,

CD4+ T lymphocyte count: (432-1341)/ μL,

CD8+ T lymphocyte count: 238-1075/ μL,

Na+: (137-147) mmol/L, CRP: (0-5) mg/L,

Monocyte counts: (0.1-0.6) * 10^9/L.

C-reactive protein: (0-5) mg/L
